# Elimination of probable praziquantel-resistant *Dipylidium caninum* with nitroscanate in a mixed-breed dog: a case report

**DOI:** 10.1186/s13071-022-05559-2

**Published:** 2022-11-22

**Authors:** John P. Loftus, Andrew Acevedo, Dwight D. Bowman, Janice L. Liotta, Timothy Wu, Melinda Zhu

**Affiliations:** 1 Department of Clinical Sciences, College of Veterinary Medicine, Cornell University, Ithaca, NY USA; 2 Department of Microbiology and Immunology, College of Veterinary Medicine, Cornell University , Ithaca, NY USA; 3 Williamsburg Animal Clinic, 760 Grand St., Brooklyn, NY 11211a USA; 4 Present Address: Center for Bird and Exotic Animal Medicine, 11401 NE 195Th St, Bothell, WA 98011 USA

**Keywords:** Praziquantel, Nitroscanate, Dipylidium, Capsule endoscopy

## Abstract

**Background:**

Praziquantel is the drug of choice for treating the tapeworm *Dipylidium caninum* in dogs; however, resistance is possible, and regular, non-targeted administration of praziquantel may select for anthelminthic-resistant populations.

**Methods:**

The zinc sulfate fecal floatation procedure was conducted. Gross visualization was used to identify *Dipylidium* spp. segments, and capsule endoscopy was used to visualize adult tapeworms within the intestinal tract.

**Results:**

An 18-month-old spayed female terrier mix was presented due to diarrhea, hematochezia and weight loss. The dog received appropriate anthelmintic therapy for *Giardia* spp., *Ancylostoma* spp. and *Dipylidium* spp. The dog’s clinical signs resolved, and elimination of *Ancylostoma* spp. was confirmed by subsequent fecal analysis. However, *Dipylidium* spp. segments were repeatedly present in the stool. Observation of the segments confirmed the presence of adult *Dipylidium* spp in feces. Treatment with praziquantel and epsiprantel were unsuccessful in eliminating the organism but was apparently successful in flea prevention. A single dose of nitrosconate was administered and eliminated *Dipylidium* spp. infection in the dog.

**Conclusions:**

Nitrosconate can be an effective treatment for praziquantel-resistant dipylidiasis in dogs. The novel application of capsule endoscopy confirmed the anthelmintic efficacy of this treatment.

**Graphical Abstract:**

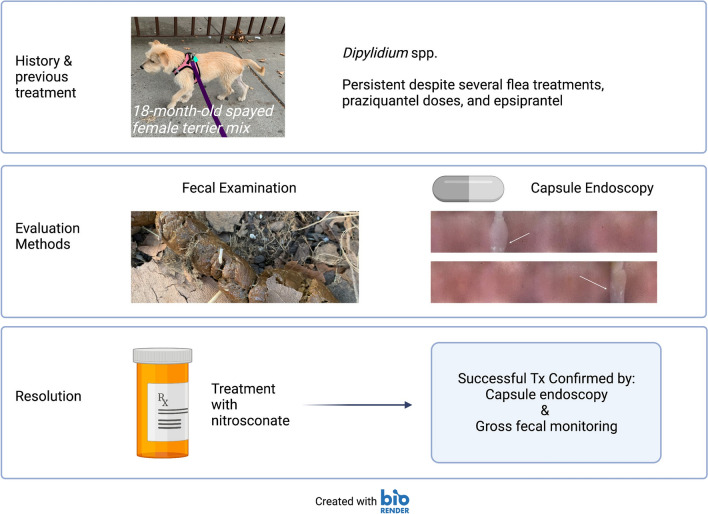

**Supplementary Information:**

The online version contains supplementary material available at 10.1186/s13071-022-05559-2.

*Dipylidium caninum* is a cyclophyllidean cestode parasite of the small intestine in dogs, with an indirect life-cycle primarily transmitted by fleas (*Ctenocephalides felis felis*, *Ctenopcephalides canis*, *Pulex irritans*) (see [1–3] for a description of its life-cycle). However, biting lice (*Trichodectes canis*) can also act as intermediate hosts. Gross visualization of cucumber seed-shaped proglottids in the stool or microscopic identification of egg packets by the fecal flotation procedure is diagnostic for infection with this parasite. However, proglottids may not be uniformly present in feces, and eggs are not always identified in the fecal analysis of infected individuals [1]. The primary treatment for tapeworm infestations in dogs and cats is praziquantel, available in oral, injectable and topical forms. Many current commercial products offer praziquantel in combination with other parasiticides. When combined with other products, praziquantel can be 100% effective for treating tapeworms in dogs [4–6]. Other treatments used in the past include epsiprantel, nitroscanate and off-label use of nitazoxanide [7].

Anthelminthic resistance is described as the phenomenon in which heritable genetic changes in a population of helminths result in a larger proportion of the population remaining alive following the administration of a previously effective drug dose [3]. The potential for the emergence of drug-resistant helminth populations has been attributed to a move away from infrequent, targeted treatments towards regular, metaphylactic treatment using broad-spectrum anthelmintics [8]. Indeed, reports of anthelminthic-resistant populations of canine parasites have emerged, including reports of resistant *Dirofilaria immitis*, *Ancylostoma caninum* and, most recently, *Dipylidium caninum* [3, 9]*.* In a 2018 report, *Dipylidium* spp. resistance to standard doses of praziquantel was described, with favorable responses to higher doses or alternative treatments, such as nitroscanate [10].

The objective of this article is to document a case of probable praziquantel-resistant *Dipylidium caninum* effectively treated with nitroscanate. We also demonstrate the utility of capsule endoscopy to monitor the effectiveness of anthelmintic therapy in a dog.

An 18-month-old spayed female terrier mix, with a body weight of 9.7 kg, was presented to her primary veterinarian with a history of chronic gastrointestinal signs and persistent *Dipylidium* spp. infection. Tapeworm segments were first found in the dog’s stool at 4 months of age, prompting evaluation by the primary veterinarian. The owners reported that she was the sole dog in the household and that they had never observed fleas in their home. The god presented with diarrhea, hematochezia and an underweight body condition score of 2/5; no evidence of fleas was noted on physical exam and from patient history. Pyrantel pamoate, metronidazole and fenbendazole were recommended for general deworming. Initial differential diagnoses included *Giardia* spp., tapeworms, hookworms, roundworms and whipworms. The initial fecal flotation procedure revealed *Ancylostoma* spp. After treatment, parasites were not detected with subsequent fecal testing (fecal ova and parasite [O&P] examination with centrifugation test, performed with zinc sulfate solution; Antech Diagnostics, Southaven, MS, USA) and *Giardia* enzyme-linked immunosorbent assay (ELISA) tests; however, the owner reported finding tapeworm segments in the stool (Fig. 1) 1 month post-treatment. Praziquantel with pyrantel pamoate (Drontal®; Bayer Animal Health) was administered. However, the organism was not eliminated, as once again the owner reported tapeworm segments in the feces at about 1 month post-treatment. The dog was then treated with milbemycin oxime (Interceptor®; Eli Lilly and Company, Indianapolis, IN, USA), afoxolaner (NexGard®; Boehringer Ingelheim, Ingelheim, Germany), imidacloprid and flumethrin (Seresto® collar; Elanco Animal Health, Greenfield, IN, USA) to control fleas as a source of reinfection, as well as with repeated doses of praziquantel with pyrantel pamoate and febantel (Drontal® Plus) (Table 1), but the infection was not eliminated. Epsiprantel (Cestex®; Zoetis, Parsippany–Troy Hills, NJ, USA) was also unsuccessful. The owners did not report evidence of a flea infestation, but it was recommended that they undertake aggressive environmental decontamination. This action included an inspection by a professional exterminator who reported no evidence of fleas and did not advise environmental treatment. However, the owners, desperate to alleviate their pet of tapeworms, used an over-the-counter “flea bomb” (Advantage® Household fogger; Elanco Animal Health) in the dog’s place of residence in the hope of disrupting the parasite life-cycle. Afterward, to limit exposure to reinfection, the dog was restricted to house confinement for approximately 6 months, with no dog park or daycare exposure. Samples of the tapeworm segments were sent to Cornell University and identified as *Dipylidium* sp. In order to establish resistance in the context of clinical case management, praziquantel, milbemycin oxime, lufenuron (Sentinel®; Merck Animal Health USA, Madison, NJ, USA) and fluralaner (Bravecto®; MSD Animal Health, Rahway, NJ, USA), were dosed regularly; nitenpyram (Capstar®; Novartis, Basel, Switzerland) was administered repeatedly; and fecal samples were frequently evaluated for the presence of tapeworm segments (Table 1). Capsule endoscopy was employed to directly visualize adult tapeworms in the small intestine. For the endoscopy procedure, the patient was first fasted for 12 h. The endoscopic capsule (CapsoCam Plus® video capsule system; CapsoVision, Inc., Saratoga, CA, USA0) was then administered orally by a trained veterinary technician, followed by 5–10 ml of water administered by a syringe. The capsule was recovered in the feces and shipped to the primary author’s institution. Acquired data were downloaded with software (CapsoView®; CapsoVision, Inc., Saratoga, CA, USA) for image review. The retrieved images disclosed adult tapeworm segments in the intestinal tract before and after nitenpyram (Capstar®) treatments (Fig. 2; Additional File 1: Video). After the last nitenpyram administration, nitrosconate (Lopatol®; Novartis) was administered (one dose, 200 mg. orally). Fecal samples collected before and after the nitrosconate treatment confirmed treatment efficacy. Capsule endoscopy was repeated after the administration of nitrosconate and corroborated the elimination of *Dipylidium* organisms. At the time of writing this paper, the dog’s primary veterinarian reports that after 18 months following the treatment, the dog remains tapeworm free and has not shed tapeworms.

**Fig. 1:**
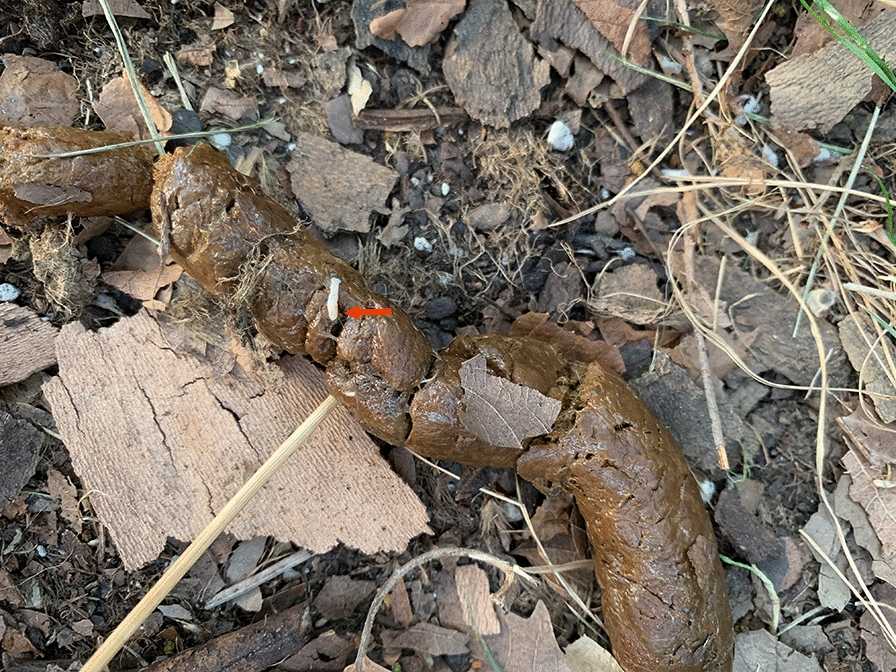
Representative images of feces containing *Dipylidium* segments. Arrow indicates tapeworm segment

**Table 1:** Summary of treatments & diagnostic evaluations

Date	Treatment	Diagnostics	Comments
11/2019 & prior	praziquantel, ^1^Drontal®, ^2^Interceptor®, ^3^Nexgard®	Fecal floatation (exact method unknown)	This was before presenting to the primary veterinarian
11/21/2019	^1^Drontal®		
12/1/2019	^4^Drontal® Plus		Segments observed
1/2020	^4^Drontal® Plus, ^5^Seresto® collar, ^6^Cestex®	Fecal O&P with Centrifugation	Segments observed
2/2020	^4^Drontal® Plus		The dog did not go to dog parks or daycare during COVID quarantine
4/24/2020	^4^Drontal® Plus		Owner observed tapeworm segments, began using a basket muzzle to source of potential unknown reinfection
5/22/2020			Owner reported exterminator visit confirming no fleas, “flea bomb” performed prophylactically. Confirmed dog is still receiving flea preventatives
6/17/2020	Praziquantel 20 mg/kg SID × 2 weeks		
6/19/2020			Live tapeworms seen in stool
7/20/2020			Cornell received tapeworm sample and confirmed identity as Dipylidium spp. New treatment protocol initiated
8/5/20	^7^Sentinel® & ^8^Bravecto®		
8/6/20	^9^Capstar®		
8/7/20	^9^Capstar®		
8/8/20	^9^Capstar®		
8/9/20	^9^Capstar®		
8/10/20	^9^Capstar®	CapsoCam	Adult tapeworm(s) present
8/11/20	^9^Capstar®		
8/12/20	^9^Capstar®		
8/13/20	^9^Capstar®		Live tapeworm recovered in stool
8/14/20	^9^Capstar® & praziquantel (5 mg/kg)		
8/15/20			
8/16/20			
8/17/20		CapsoCam	Adult tapeworm(s) present
9/18/2020	^10^Lopatol® (100 mg/kg once)		
10/7/2020		CapsoCam	No tapeworms present
11/6/2020		CapsoCam	No tapeworms present
6/2/2020		CapsoCam	No tapeworms present; no segments reported by owner after Lopatol treatment

^1^Praziquantel with pyrantel pamoate

^2^Milbemycin oximine

^3^Afoxolaner

^4^ Praziquantel with pyrantel pamoate and febantel

^5^Imidacloprid and flumethrin

^6^Epsiprantel

^7^Lufenuron

^8^Fluralaner

^9^Nitenpyram

^10^Nitrosconate

**Fig. 2:**
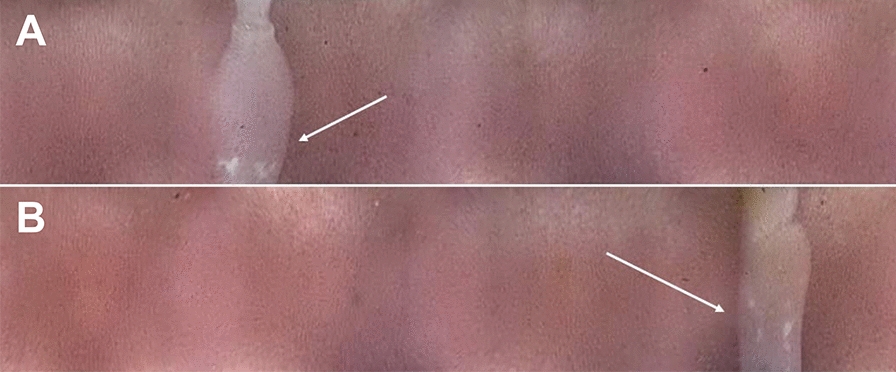
Capsule endoscopy images. Two (**A** &** B**) representative images of *Dipylidium* (indicated by arrows) in the small intestine before treatment with nitrosconate. The capsule endoscope used in this case employs side-facing cameras. Images are panoramic (360) views of the intestinal mucosa

This report documents probable praziquantel-resistant *Dipylidium* sp. that was effectively treated with nitrosconate and highlights the novel use of capsule endoscopy for assessing parasite burden. Throughout this case report, the efficacy of anthelmintic therapy was evaluated by repeated testing for the presence of parasites. Monitoring the stool, grossly and through fecal flotation and *Giardia* ELISA, ruled out other differential diagnoses and assessed if treatment effectively resolved the *Dipylidium* sp. infection. Additionally, capsule endoscopy provided direct visualization of adult tapeworm scolices with segments attached to the small intestinal mucosa and proglottids. In this case, diagnosis of the resistant parasite was based on the presence of proglottids in the stool and on capsule endoscopy following treatment with praziquantel. However, subclinical infections can occur, in which helminths that are not easily visualized grossly (such as with anthelminthic-resistant *Ancylostoma caninum*); therefore, it is important for the veterinarian to routinely monitor for active parasite infections and effective treatment utilizing pre- and post-treatment coproparasitological techniques, such as fecal flotation [11].

Limitations of this case report include the authors’ inability to fully assess the efficacy of environmental decontamination done at the patient's home. Flea infestations are difficult to eliminate in a household, especially when the infestation source, usually a pet, continually re-introduces fleas. Although an over-the-counter “flea bomb” was used, it is a possibility that insufficient environmental decontamination may confound the repeated appearance of tapeworm segments in the stool. However, the aggressive course of flea treatment, environmental decontamination, and evaluation by a professional exterminator make re-infection extremely unlikely in this case. Additionally, the extensive flea treatments and monitoring conducted relative to the last dose of praziquantel makes reinfection as explanation for tapeworm persistence nearly impossible in this case. Although helpful in visualizing the small intestine, images obtained from capsule endoscopy were occasionally obscured by intestinal content, making visualization difficult.

*Dipylidium caninum* is the most common tapeworm in dogs and cats, with a prevalence ranging from 4.0% to 60.0% [7], with some reports as low as 0.3% [12]. The recommended treatment of this tapeworm is 5 mg/kg of praziquantel [6]; however, the exact dosage often varies, as products sold contain fixed amounts of the drug and are labeled for specific weight ranges. This drug tends to have mild to no adverse effects (anorexia, vomiting, lethargy, and diarrhea, which are usually associated with higher doses). It may be given at higher doses or repeated weekly or even daily to treat some infections. One study found that dogs treated with six times the recommended therapeutic dose vomited only occasionally [4]. Typically, *Dipylidium caninum* infections cause little to no clinical signs in dogs and cats. On the rare occasion that dipylidiasis causes clinical signs, abdominal pain, vomiting, diarrhea, and mild anal pruritus may be encountered. In this case, we chose nitrosconate over nitazoxanide due to availability and the lower cost of the former. The safety of nitrosconate has also been previously reported in dogs [13]. However, since treating this case, the authors have learned that nitrosconate may no longer be available, and in some cases, nitazoxanide may be the only viable treatment for cases that do not respond to the first or second-line treatments described above.

Several challenges were encountered in this case. In tapeworm infections involving flea infestations, re-infection is common, as fleas continually hatch in the environment, and a patient may ingest these new fleas after treatment. Thus, praziquantel resistance may be mistakenly assigned to situations where the infection was only temporarily eliminated. Additionally, there is a pre-patent period of approximately 2.5 weeks after infection before dogs and cats begin to shed proglottids that can be seen in feces [7].

This case corroborates the potential efficacy of nitrosconate for treating praziquantel-resistant *Dipylidium caninum*. Additionally, capsule endoscopy can assist in confirming anthelmintic treatment success in infections where fecal analysis alone may not adequately demonstrate the elimination of organisms.

## Supplementary Information

**Additional file 1**. Video: Capsule endoscopy video of a tapeworm in the dog’s small intestine. This movie captures the tapeworm segment in the small intestine of the dog in this case report before nitrosconate administration. This is a side-facing camera capsule endoscope, therefore the images depict a panoramic (360 deg) view of the the small intestinal mucosa.

## Data Availability

Data are available upon request, with patient identifiers redacted.
