# Correction: Elimination of probable praziquantel-resistant *Dipylidium caninum* with nitroscanate in a mixed-breed dog: a case report

**DOI:** 10.1186/s13071-023-05716-1

**Published:** 2023-03-07

**Authors:** John P. Loftus, Andrew Acevedo, Dwight D. Bowman, Janice L. Liotta, Timothy Wu, Melinda Zhu

**Affiliations:** 1 Department of Clinical Sciences, College of Veterinary Medicine, Cornell University, Ithaca, NY USA; 2 Department of Microbiology and Immunology, College of Veterinary Medicine, Cornell University, Ithaca, NY USA; 3 Williamsburg Animal Clinic, 760 Grand St, Brooklyn, NY 11211a USA; 4 Present Address: Center for Bird and Exotic Animal Medicine, 11401 NE 195Th St, Bothell, WA 98011 USA

**Correction: Parasites & Vectors (2022) 15:438** **https://doi.org/10.1186/s13071-022-05559-2**

Following publication of the original article [1], the authors flagged errors in the reported doses of Lopatol® (nitrosconate): in the main body of the article, a 200 mg dose had been stated in place of 500 mg, the correct dose; in Table 1, a 100 mg/kg dose had been reported in place of a 50 mg/kg dose, the correct dose.

The errors have since been corrected in the published article. The authors thank you for reading this correction and apologize for any inconvenience caused.
